# Supplementary material for: National Burden and Trends for 29 Groups of Cancer in Mexico from 1990 to 2019: A Secondary Analysis of the Global Burden of Disease Study 2019
Source: Cancers (Basel). 2023 Dec 28;16(1):149. doi: 10.3390/cancers16010149 (PMC10778521; doi:10.3390/cancers16010149)
Supplement: Supplementary file 1 [file cancers-16-00149-s001.zip › cancers-2762340-supplementary.pdf]

Supplementary Information

Supplementary Figure S1. Cancer-specific trends and annual percentage change from 1990 to 2019 of the age-standardized incidence rates (per 100,000 people) in Mexico. 2

Supplementary Figure S2. Cancer-specific annual percentage changes from 1990 to 2019 of age-standardized incidence (a) and mortality (b) rates per 100,000 population in Mexico. 3

Supplementary Figure S3. Cancer-specific incidence rates (a) and age-standardized mortality rates (per 100,000 people) by sex (b) in Mexico in 2019. 4

Supplementary Figure S4. Age-specific incidence rates (per 100,000 people) by sex for each cancer group in Mexico during 2019...5

Supplementary Table S1. Total and sex-specific and age-standardized incidence and mortality rates (per 100,000 people) by cancer group in Mexico during 2019...6

Supplementary Table S2. Percentage change from 1990 to 2019 in crude incidence and mortality due to 29 malignant neoplasms in Mexico...8

Supplementary Table S3. Percentage change from 1990 to 2019 of age-standardized incidence and mortality rates (per 100,000 population) due to 29 malignant neoplasms in Mexico. 10

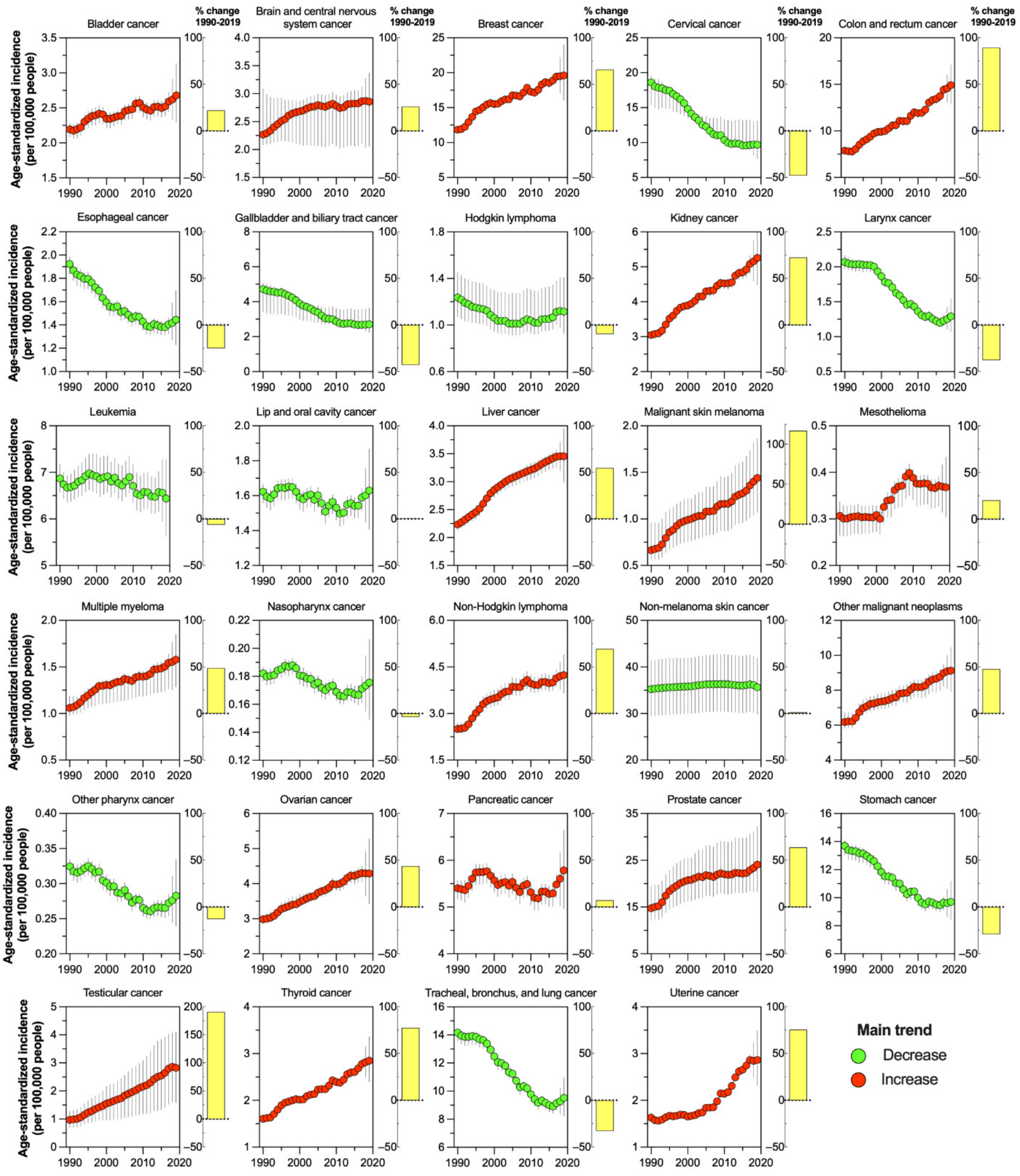

Supplementary Figure S1. Cancer-specific trends and annual percentage change from 1990 to 2019 of the age-standardized incidence rates (per 100,000 people) in Mexico.

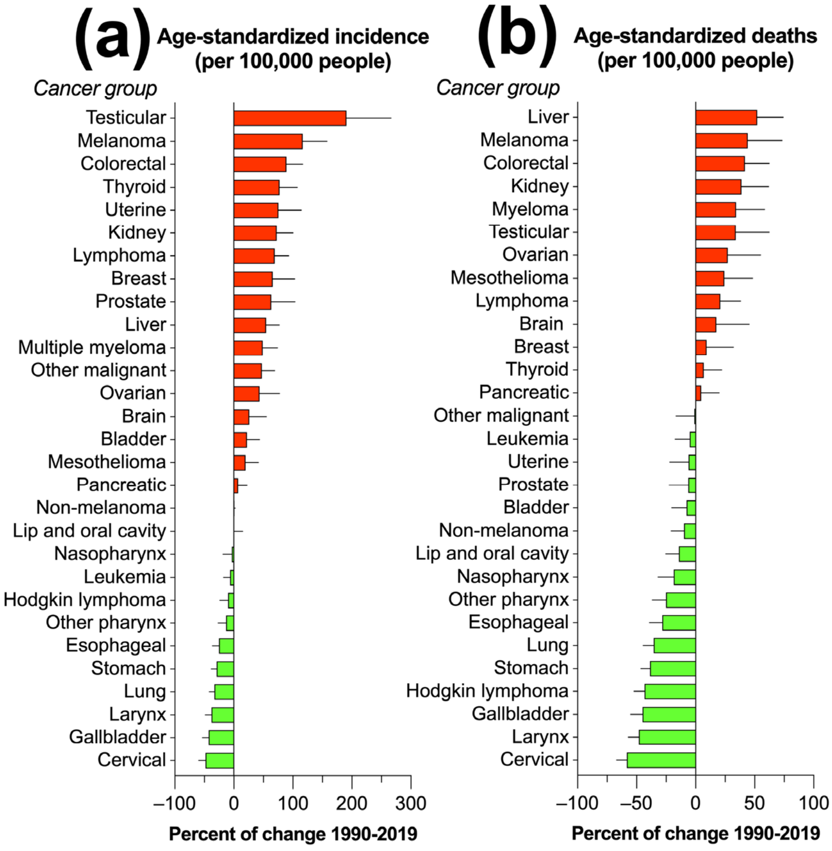

Supplementary Figure S2. Cancer-specific annual percentage changes from 1990 to 2019 of age-standardized incidence (a) and mortality (b) rates per 100,000 population in Mexico.

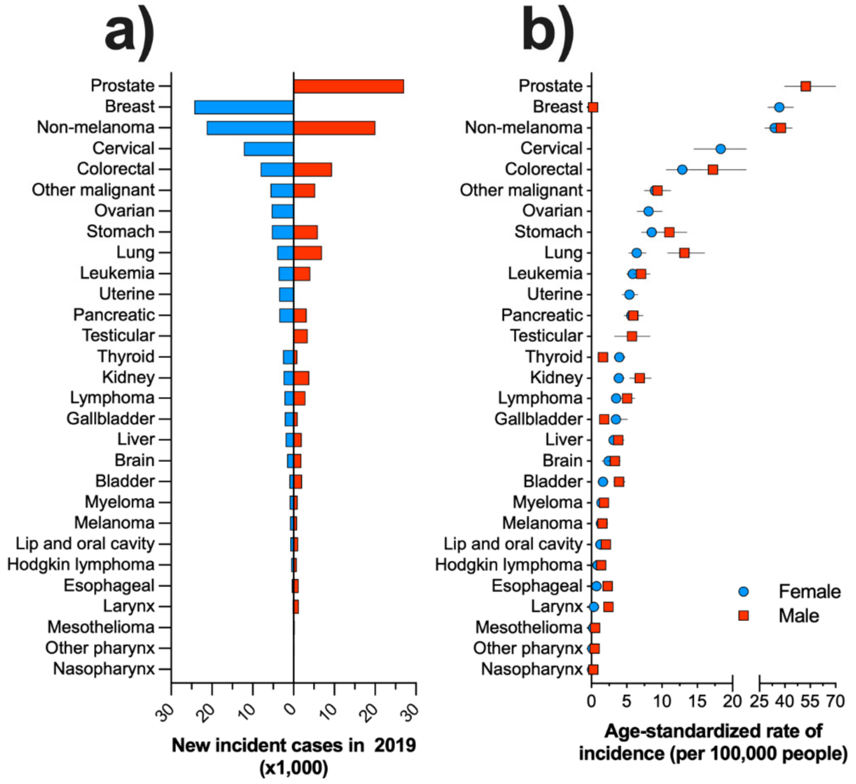

Supplementary Figure S3. Cancer-specific incidence rates (a) and age-standardized mortality rates (per 100,000 people) by sex (b) in Mexico in 2019.

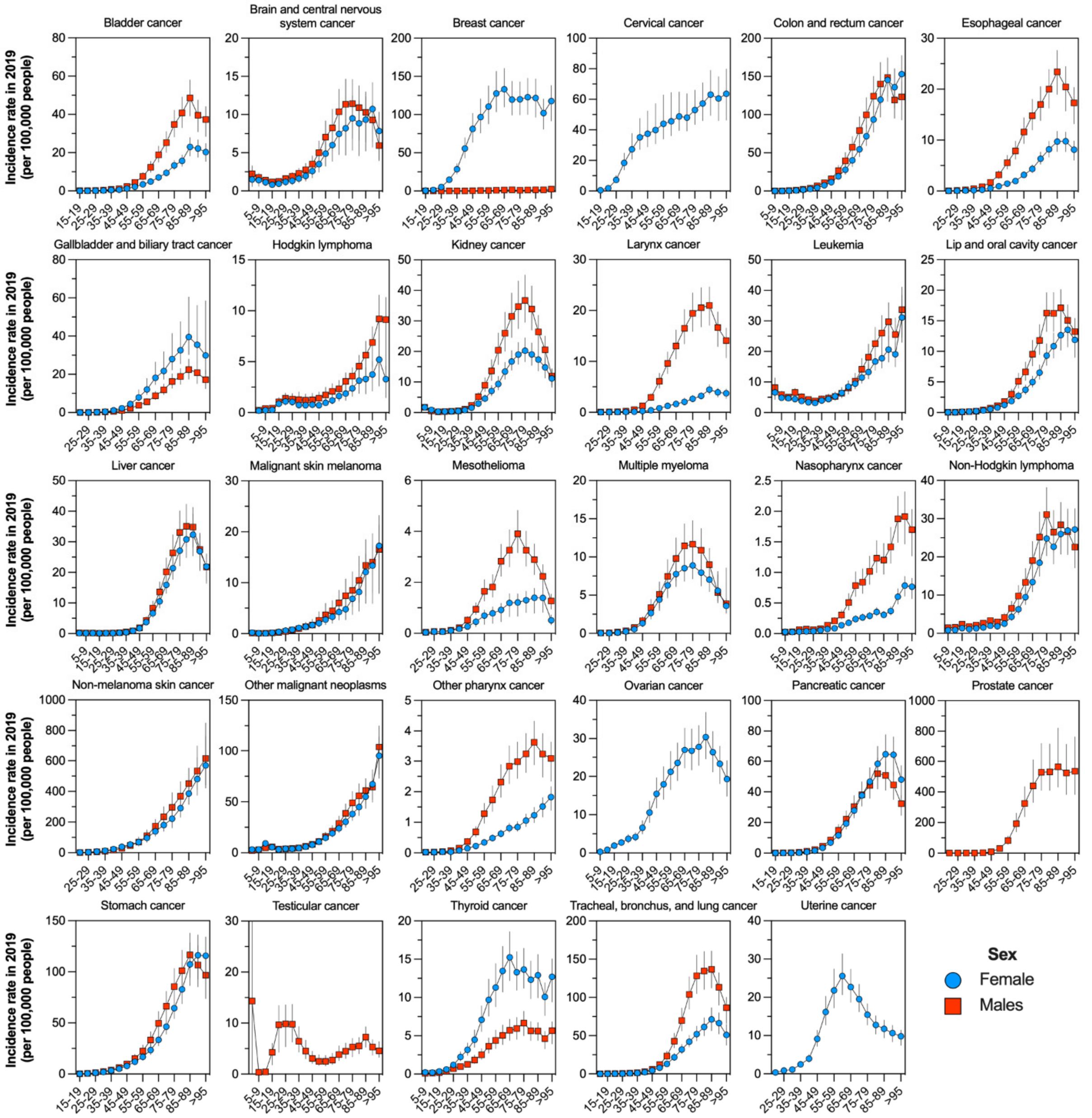

Supplementary Figure S4. Age-specific incidence rates (per 100,000 people) by sex for each cancer group in Mexico during 2019.

Supplementary Table S1. Total and sex-specific and age-standardized incidence and mortality rates (per 100,000 people) by cancer group in Mexico during 2019.

Group of cancer	Incidence (per 100,000 people: 95% UI)			Deaths (per 100,000 people: 95% UI)		
	Total	Female	Male	Total	Female	Male
Bladder cancer	2.68 (2.30 to 3.13)	1.64 (1.36 to 1.98)	3.90 (3.17 to 4.76)	1.52 (1.30 to 1.76)	0.96 (0.80 to 1.16)	2.19 (1.80 to 2.63)
Brain and central nervous system cancer	2.86 (2.06 to 3.37)	2.44 (1.50 to 3.08)	3.31 (2.31 to 4.07)	2.31 (1.68 to 2.72)	1.94 (1.19 to 2.44)	2.72 (1.90 to 3.36)
Breast cancer	19.62 (16.03 to 23.99)	36.81 (30.02 to 45.04)	0.23 (0.19 to 0.29)	6.72 (5.59 to 8.14)	12.48 (10.35 to 15.16)	0.14 (0.11 to 0.17)
Cervical cancer	9.68 (7.68 to 13.11)	18.34 (14.55 to 24.83)	-	5.08 (4.10 to 6.74)	9.53 (7.68 to 12.64)	-
Colon and rectum cancer	14.92 (12.88 to 17.11)	12.90 (10.60 to 15.63)	17.22 (14.15 to 21.16)	9.24 (7.95 to 10.52)	8.09 (6.70 to 9.66)	10.55 (8.69 to 12.73)
Esophageal cancer	1.44 (1.23 to 1.69)	0.72 (0.60 to 0.87)	2.28 (1.85 to 2.78)	1.51 (1.28 to 1.75)	0.75 (0.62 to 0.90)	2.40 (1.93 to 2.91)
Gallbladder and biliary tract cancer	2.71 (2.25 to 3.64)	3.46 (2.76 to 5.09)	1.83 (1.44 to 2.31)	2.57 (2.14 to 3.43)	3.28 (2.59 to 4.83)	1.74 (1.35 to 2.20)
Hodgkin lymphoma	1.11 (0.93 to 1.41)	0.88 (0.67 to 1.33)	1.37 (1.09 to 1.75)	0.53 (0.44 to 0.67)	0.40 (0.30 to 0.57)	0.67 (0.53 to 0.85)
Kidney cancer	5.26 (4.48 to 6.13)	3.87 (3.22 to 4.66)	6.85 (5.44 to 8.46)	2.97 (2.51 to 3.46)	2.07 (1.72 to 2.49)	4.01 (3.15 to 4.99)
Larynx cancer	1.29 (1.08 to 1.54)	0.35 (0.28 to 0.47)	2.39 (1.93 to 2.91)	0.99 (0.82 to 1.18)	0.27 (0.22 to 0.36)	1.84 (1.49 to 2.22)
Leukemia	6.44 (5.63 to 7.28)	5.88 (4.98 to 6.93)	7.04 (5.96 to 8.29)	4.37 (3.84 to 4.95)	3.88 (3.30 to 4.56)	4.92 (4.11 to 5.85)
Lip and oral cavity cancer	1.63 (1.41 to 1.87)	1.26 (1.05 to 1.52)	2.05 (1.67 to 2.49)	0.98 (0.84 to 1.11)	0.72 (0.60 to 0.87)	1.27 (1.05 to 1.53)
Liver cancer	3.46 (2.99 to 3.96)	3.15 (2.62 to 3.80)	3.81 (3.12 to 4.62)	3.69 (3.18 to 4.22)	3.37 (2.79 to 4.05)	4.06 (3.31 to 4.92)
Malignant skin melanoma	1.44 (1.07 to 1.86)	1.33 (0.80 to 1.75)	1.57 (0.98 to 2.28)	0.76 (0.54 to 1.00)	0.62 (0.36 to 0.81)	0.92 (0.57 to 1.36)

Mesothelioma	0.37 (0.30 to 0.43)	0.23 (0.14 to 0.29)	0.53 (0.43 to 0.65)	0.30 (0.25 to 0.35)	0.18 (0.11 to 0.23)	0.44 (0.35 to 0.53)
Multiple myeloma	1.58 (1.28 to 1.85)	1.41 (1.10 to 1.73)	1.77 (1.26 to 2.25)	1.24 (0.99 to 1.47)	1.06 (0.85 to 1.31)	1.43 (1.02 to 1.83)
Nasopharynx cancer	0.18 (0.15 to 0.21)	0.10 (0.08 to 0.12)	0.27 (0.21 to 0.33)	0.15 (0.12 to 0.17)	0.07 (0.06 to 0.09)	0.23 (0.19 to 0.29)
Non-Hodgkin lymphoma	4.24 (3.66 to 4.90)	3.50 (2.89 to 4.28)	5.05 (4.13 to 6.14)	2.90 (2.51 to 3.30)	2.45 (2.04 to 2.96)	3.40 (2.78 to 4.14)
Non-melanoma skin cancer	35.66 (29.80 to 41.90)	34.01 (28.19 to 40.22)	37.69 (31.67 to 44.20)	1.21 (1.03 to 1.39)	1.03 (0.85 to 1.22)	1.43 (1.15 to 1.73)
Other malignant neoplasms	9.13 (7.92 to 10.47)	8.98 (7.53 to 10.70)	9.36 (7.61 to 11.24)	4.41 (3.78 to 5.08)	3.95 (3.25 to 4.75)	4.94 (3.97 to 6.00)
Other pharynx cancer	0.28 (0.24 to 0.33)	0.14 (0.12 to 0.17)	0.45 (0.36 to 0.55)	0.24 (0.21 to 0.28)	0.11 (0.09 to 0.13)	0.40 (0.32 to 0.48)
Ovarian cancer	4.29 (3.43 to 5.28)	8.12 (6.50 to 10.00)	-	2.78 (2.24 to 3.42)	5.21 (4.21 to 6.42)	-
Pancreatic cancer	5.78 (4.97 to 6.64)	5.63 (4.64 to 6.81)	5.94 (4.79 to 7.28)	6.01 (5.16 to 6.87)	5.88 (4.88 to 7.07)	6.13 (4.98 to 7.41)
Prostate cancer	24.09 (18.39 to 32.20)	-	52.35 (40.04 to 70.09)	8.78 (6.69 to 12.08)	-	19.37 (14.77 to 26.70)
Stomach cancer	9.70 (8.43 to 11.19)	8.53 (7.10 to 10.27)	11.05 (9.11 to 13.51)	8.86 (7.64 to 10.16)	7.64 (6.35 to 9.17)	10.28 (8.45 to 12.41)
Testicular cancer	2.83 (1.58 to 4.11)	-	5.76 (3.29 to 8.28)	0.52 (0.41 to 0.69)	-	1.08 (0.84 to 1.41)
Thyroid cancer	2.85 (2.40 to 3.36)	3.94 (3.20 to 4.81)	1.62 (1.29 to 2.00)	0.83 (0.71 to 0.95)	1.04 (0.86 to 1.25)	0.58 (0.46 to 0.71)
Tracheal, bronchus, and lung cancer	9.52 (8.23 to 10.97)	6.41 (5.30 to 7.74)	13.17 (10.84 to 16.05)	9.74 (8.36 to 11.24)	6.27 (5.11 to 7.62)	13.83 (11.31 to 16.66)
Uterine cancer	2.86 (2.32 to 3.49)	5.40 (4.37 to 6.58)	-	0.79 (0.65 to 0.96)	1.47 (1.22 to 1.79)	-

95% UI: 95% Uncertainty intervals

Supplementary Table S2. Percentage change from 1990 to 2019 in crude incidence and mortality due to 29 malignant neoplasms in Mexico.

Group of cancer	Incidence (% of change 1990-2019, 95% UI)	Deaths (% of change 1990-2019, 95% UI)
Bladder cancer	246 (195 to 308)	174 (135 to 218)
Brain and central nervous system cancer	129 (49 to 181)	134 (54 to 189)
Breast cancer	317 (238 to 411)	185 (135 to 246)
Cervical cancer	27 (-1 to 97)	10 (-13 to 77)
Colon and rectum cancer	421 (348 to 498)	304 (251 to 365)
Esophageal cancer	112 (80 to 149)	107 (75 to 144)
Gallbladder and biliary tract cancer	61 (31 to 142)	57 (28 to 134)
Hodgkin lymphoma	70 (44 to 107)	19 (0 to 41)
Kidney cancer	291 (233 to 356)	256 (197 to 316)
Larynx cancer	75 (45 to 110)	48 (22 to 76)
Leukemia	34 (17 to 53)	59 (37 to 81)
Lip and oral cavity cancer	170 (134 to 210)	142 (110 to 178)
Liver cancer	310 (254 to 371)	314 (257 to 376)
Malignant skin melanoma	436 (264 to 541)	283 (147 to 361)
Mesothelioma	210 (158 to 267)	227 (173 to 291)
Multiple myeloma	306 (239 to 374)	270 (205 to 336)
Nasopharynx cancer	137 (101 to 180)	109 (75 to 147)
Non-Hodgkin lymphoma	239 (191 to 289)	170 (133 to 210)
Non-melanoma skin cancer	175 (168 to 181)	162 (127 to 200)
Other malignant neoplasms	175 (135 to 216)	113 (80 to 147)
Other pharynx cancer	140 (102 to 183)	110 (77 to 147)
Ovarian cancer	236 (165 to 316)	232 (164 to 304)
Pancreatic cancer	199 (158 to 242)	196 (155 to 240)

Prostate cancer	385 (291 to 506)	192 (141 to 255)
Stomach cancer	100 (74 to 129)	77 (55 to 105)
Testicular cancer	312 (170 to 493)	132 (69 to 189)
Thyroid cancer	338 (268 to 415)	196 (154 to 240)
Tracheal, bronchus, and lung cancer	86 (61 to 114)	82 (57 to 109)
Uterine cancer	377 (284 to 483)	169 (122 to 226)

95% UI: 95% Uncertainty intervals

Supplementary Table S3. Percentage change from 1990 to 2019 of age-standardized incidence and mortality rates (per 100,000 population) due to 29 malignant neoplasms in Mexico.

Group of cancer	Incidence (% of change 1990-2019, 95% UI)	Deaths (% of change 1990-2019, 95% UI)
Bladder cancer	22.06 (4.41 to 43.68)	-7.64 (-20.23 to 7.13)
Brain and central nervous system cancer	26.00 (-19. to 55.08)	-17.4 (-24.02 to 45.4)
Breast cancer	65.80 (34.4 to 102.9)	-9.27 (-9.54 to 32.0)
Cervical cancer	-47.9 (-59. to -17.3)	-58.2 (-67.02 to -30.7)
Colon and rectum cancer	89.22 (63.0 to 116.7)	-41.7 (-23.61 to 62.3)
Esophageal cancer	-24.9 (-36. to -12.1)	-28.2 (-39.18 to -15.6)
Gallbladder and biliary tract cancer	-42.6 (-53. to -13.6)	-44.9 (-55.07 to -18.1)
Hodgkin lymphoma	-9.78 (-23. to 9.287)	-43.3 (-52.26 to -33.1)
Kidney cancer	72.40 (47.2 to 100.0)	-38.8 (-16.60 to 61.9)
Larynx cancer	-37.6 (-48. to -25.2)	-48.1 (-56.92 to -38.5)
Leukemia	-6.29 (-17. to 6.600)	-4.89 (-17.32 to 8.29)
Lip and oral cavity cancer	0.431 (-12. to 14.84)	-14.1 (-25.30 to -1.94)
Liver cancer	54.60 (33.4 to 77.15)	-52.0 (-31.53 to 74.3)
Malignant skin melanoma	116.8 (48.8 to 157.9)	-44.2 (-6.55 to 73.2)
Mesothelioma	20.07 (0.45 to 41.67)	-24.2 (-3.96 to 48.2)
Multiple myeloma	48.76 (23.1 to 74.08)	-34.3 (-9.56 to 58.3)
Nasopharynx cancer	-3.63 (-18. to 13.57)	-18.5 (-31.87 to -4.21)
Non-Hodgkin lymphoma	69.23 (47.1 to 93.10)	-20.9 (-5.03 to 38.0)
Non-melanoma skin cancer	1.194 (-0.0 to 2.446)	-9.73 (-20.82 to 2.86)
Other malignant neoplasms	47.69 (26.3 to 69.39)	-0.97 (-16.52 to 14.5)
Other pharynx cancer	-12.8 (-26. to 2.243)	-25.0 (-36.66 to -12.3)
Ovarian cancer	43.68 (13.1 to 77.44)	-27.3 (-1.47 to 55.1)
Pancreatic cancer	7.057 (-7.6 to 22.42)	-4.63 (-9.69 to 19.9)

Prostate cancer	63.61 (32.7 to 103.5)	-6.27 (-22.35 to 12.8)
Stomach cancer	-29.1 (-38. to -19.0)	-38.7 (-46.25 to -29.4)
Testicular cancer	190.6 (91.6 to 266.2)	-34.0 (-4.59 to 62.4)
Thyroid cancer	77.29 (49.6 to 107.6)	-6.86 (-7.70 to 22.2)
Tracheal, bronchus, and lung cancer	-32.8 (-41. to -22.9)	-35.5 (-44.36 to -26.2)
Uterine cancer	75.48 (41.5 to 114.1)	-5.86 (-21.80 to 13.6)

95% UI: 95% Uncertainty intervals
